# Metabolic and Molecular Response to High-Fat Diet Differs between Rats with Constitutionally High and Low Serotonin Tone

**DOI:** 10.3390/ijms24032169

**Published:** 2023-01-21

**Authors:** Petra Baković, Maja Kesić, Darko Kolarić, Jasminka Štefulj, Lipa Čičin-Šain

**Affiliations:** 1 Department of Molecular Biology, Ruđer Bošković Institute, HR-10000 Zagreb, Croatia; 2 Centre for Informatics and Computing, Ruđer Bošković Institute, HR-10000 Zagreb, Croatia

**Keywords:** serotonin, diet-induced obesity, glucose tolerance, insulin resistance, high-fat diet, hypothalamus, adipose tissue, energy balance, thermogenesis

## Abstract

Maintaining energy balance is a complex physiological function whose dysregulation can lead to obesity and associated metabolic disorders. The bioamine serotonin (5HT) is an important regulator of energy homeostasis, with its central and peripheral pools influencing energy status in opposing ways. Using sublines of rats with constitutionally increased (high-5HT) or decreased (low-5HT) whole-body 5HT tone, we have previously shown that under standard diet constitutionally higher 5HT activity is associated with increased body weight, adiposity, and impaired glucose homeostasis. Here, we investigated the response of 5HT sublines to an obesogenic diet. Consistent with previous findings, high-5HT animals fed a standard diet had poorer metabolic health. However, in response to a high-fat diet, only low-5HT animals increased body weight and insulin resistance. They also showed more pronounced changes in blood metabolic parameters and the expression of various metabolic genes in hypothalamus and adipose tissue. On the other hand, high-5HT animals appeared to be protected from major metabolic disturbances of the obesogenic diet. The results suggest that constitutionally low 5HT activity is associated with higher susceptibility to harmful effects of a high-energy diet. High-5HT subline, which developed less adverse metabolic outcomes on hypercaloric diets, may prove useful in understanding metabolically healthy obesity in humans.

## 1. Introduction

The prevalence of obesity, a global health epidemic nowadays, has tripled worldwide in the last four decades. Despite this dramatic increase and the close association of obesity with several life-threatening comorbidities, there is still no effective drug treatment for this condition [1]. Therefore, a better understanding of the pathophysiological basis of obesity and its associated comorbidities is essential.

Serotonin (5-hydroxytryptamine, 5HT) is a biogenic monoamine widely distributed in the body. It is synthesized in the brain, by brainstem raphe neurons, where it acts as a neurotransmitter/neuromodulator, and in the periphery, mainly by enterochromaffin cells of the gut, from which it is released into the bloodstream, taken up by platelets, and transported to various organs, acting thus as an endocrine signaling molecule [2,3]. In addition, 5HT produced locally in several peripheral organs acts as a paracrine or autocrine signal. The blood-brain barrier, which is impermeable for 5HT, ensures that its central and peripheral pools are functionally separated.

Serotonin is involved in the regulation of a variety of biological processes, at both central and peripheral levels, and the dysregulation of both 5HT systems has been associated with the development of many pathophysiological conditions [4]. Further, 5HT exerts its biological functions by interacting with at least 14 different plasma membrane-bound 5HT receptor subtypes that relay signals downstream through different intracellular pathways [5], as well as by receptor-independent mechanism of serotonylation, a covalent binding of 5HT to cytoplasmic [6] and histone proteins [7]. New pathways for 5HT signaling are constantly being uncovered [8].

It is well known that 5HT plays an important function in maintaining of energy homeostasis. The role of brain 5HT system in the regulation of food intake and body weight has been studied for decades [9,10,11]. Feeding behavior is regulated by two major brain pathways–the homeostatic, associated mainly with the hypothalamus, and the hedonic, associated with the corticolimbic regions–and 5HT signaling contributes to both of these circuits by acting to reduce food intake [11,12]. Based on animal and clinical studies showing that reduced 5HT activity in the brain is associated with the development of obesity, increasing brain 5HT signaling has been proposed as a potential anti-obesity treatment [1,13,14].

The role of peripheral serotonin in regulating energy homeostasis was discovered years later [15,16,17,18]. It soon became clear that the effect of peripheral 5HT on metabolic homeostasis is opposite to that of centrally acting amine, i.e., increase in peripheral 5HT signaling is associated with increased adiposity [19,20]. Peripheral 5HT acting on energy homeostasis originates from the gut and from metabolic organs that can synthesize 5HT, such as adipose tissue and pancreas [21,22]. It contributes to energy homeostasis by stimulating adipogenesis [23,24] and lipogenesis [24,25], promoting insulin secretion [26,27], stimulating liver gluconeogenesis [28], and attenuating thermogenic activity of brown adipose tissue [24,29]. Accordingly, reducing peripheral 5HT signaling has been proposed as a novel strategy to treat obesity [3,18,19,29].

To date, several drugs aimed to either increase the activity of the central 5HT system or decrease the activity of the peripheral 5HT system have been tested for the treatment of obesity, but none has been largely successful [1,18,30]. Given the opposing functions of central and peripheral 5HT in regulating energy balance, this is not very surprising. The activity of 5HT in both compartments depends on the action of a number of 5HT-related proteins, of which the membrane 5HT transporter (5HTT, Sert), that removes 5HT from the extracellular space, plays the most important role in regulating the bioavailability of the amine [31]. Furthermore, the net effect of 5HT on energy balance depends on the cooperation of various 5HT-related proteins in different brain circuits (hypothalamic, corticolimbic, brainstem), different metabolic organs with (pancreas, adipose tissue) or without (liver, skeletal muscle) the ability to synthesize their own 5HT, the gut as the main source of circulating 5HT, and the platelets that transport 5HT to metabolic organs. It is therefore clear that serotonergic regulation of energy homeostasis is extremely complex at the level of the whole organism, and in this context in vivo studies on animal models could be of great benefit.

Using a genetic model of rats with constitutionally altered 5HT homeostasis (Wistar-Zagreb 5HT, WZ-5HT rats) we have previously shown that constitutional 5HT tone is a potent endogenous determinant of energy balance [32,33]. Two sublines of the WZ-5HT rat model, the high-5HT and low-5HT sublines, were developed by selective breeding of rats towards the extremes of platelet 5HT level and platelet 5HTT activity [34,35,36,37]. Further neurochemical, pharmacological and behavioral studies showed that genetic selection for platelet 5HT parameters resulted in differences in the functioning of both the peripheral and central 5HT systems, with animals from the high-5HT subline having constitutionally higher whole-body 5HT tone than animals from the low-5HT subline [37,38,39,40,41,42]. On a standard diet, high 5HT animals exhibited higher body weight, increased adiposity and impaired glucose homeostasis compared to low 5HT animals [32,33].

The development of obesity is the result of the interaction between genetic and lifestyle factors, with diet being one of the most important determinants [43]. In many preclinical studies investigating the etiopathogenesis of obesity, a high-fat diet (HFD) is used as an experimental protocol to create a so-called diet-induced obesity (DIO) model [44,45,46,47]. In the present study, we used HFD approach to tackle the role of constitutional 5HT tone in regulating response to metabolic challenge triggered by an obesogenic diet. Thus, we compared HFD-induced functional and molecular changes between our sublines of rats with constitutionally different 5HT homeostasis and basal energy balance. In particular, we compared their body weight gain, food intake, insulin resistance, thermogenic activity, blood metabolic parameters and the expression of various metabolic genes in the brain (hypothalamus) and in the periphery (adipose tissue).

## 2. Results

### 2.1. Characteristics of the Study Animals

**Figure 1:**
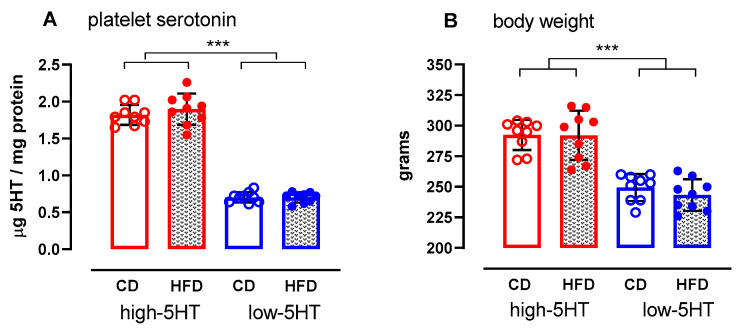
Platelet serotonin (5HT) levels (**A**) and body weight (**B**) in high-5HT and low-5HT animals used in the study. Platelet serotonin level was determined two weeks before initiation of the controlled feeding while body weight was monitored for one week before initiation of the controlled feeding. Data are presented as individual values and means ± SD, N = 9 per group; *** *p* < 0.0001 (LSD post-hoc test after one-way ANOVA), CD = control diet; HFD = high-fat diet.

The characteristics of high-5HT and low-5HT animals randomly assigned to either a standard, control diet (CD) or a high-fat diet (HFD) group are shown in Figure 1. Consistent with our previous studies, the high-5HT animals had approximately 100% higher platelet 5HT content than the low-5HT animals. At the beginning of the experiment, they had approximately 15% higher body weight. The values of both parameters did not differ between the CD and HFD groups of each 5HT subline.

### 2.2. Food Intake

**Figure 2:**
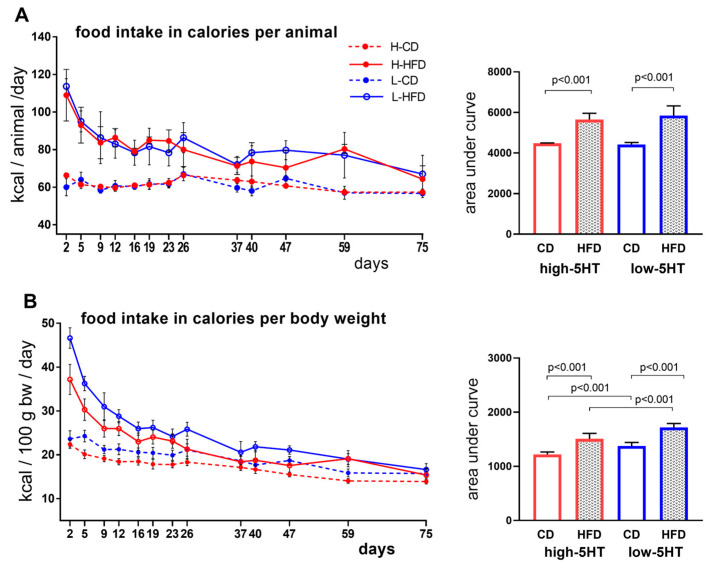
Daily food intake in animals from 5HT sublines over 11 weeks of feeding with control diet (CD) or high–fat diet (HFD), expressed in calories per animal (**A**) or calories per body weight (**B**). Corresponding area under curve (AUC) values are shown on the right. Data are presented as means ± SD; N = 9 per group; *p*-values obtained by LSD post-hoc test after one-way ANOVA. H-CD = animals from high-5HT subline on control diet; L-CD = animals from low-5HT subline on control diet; H-HFD = animals from high-5HT subline on high-fat diet; L-HFD = animals from low-5HT subline on high-fat diet.

A schematic illustrating the experimental design is provided in the Materials and Method section. Briefly, at the age of 2.5 months, rats from the CD group of each 5HT subline continued with a standard chow diet while animals from the HFD groups were fed HFD for the following 11 weeks. The monitoring of daily food intake showed that in both 5HT sublines, the mass of high-fat food consumed per animal was lower than that of the control food (Figure S1A), while caloric intake was higher in the HFD groups than in the CD groups, as expected (Figure 2A). Similar differences were observed between the HFD and CD groups when food intake was normalized to body weight (Figure 2B and Figure S1B).

### 2.3. Body/Organ Mass Changes

**Figure 3:**
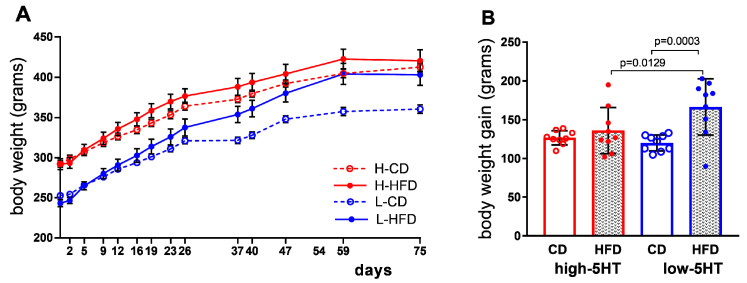
Body weight accumulation (**A**) and total body weight gain (**B**) over 11 weeks of feeding with control diet (CD) or high–fat diet (HFD). Data presented as means ± SEM (**A**) or means ± SD (**B**); N = 9 per group; *p*-values obtained by LSD post-hoc test after one-way ANOVA are shown. H-CD = animals from high-5HT subline on control diet; L-CD = animals from low-5HT subline on control diet; H-HFD = animals from high-5HT subline on high-fat diet; L-HFD = animals from low-5HT subline on high-fat diet.

In the low-5HT animals, the differences in body weight between the HFD and CD groups gradually increased, and by the end of week 11, their body weight differed by 12% (*p* = 0.008; Figure 3A), while their weight gain differed by 38% (*p* = 0.002; Figure 3B). In contrast, in the high-5HT animals, the differences in body weight (Figure 3A) and weight gain (Figure 3B) between the HFD and CD groups were not statistically significant.

**Figure 4:**
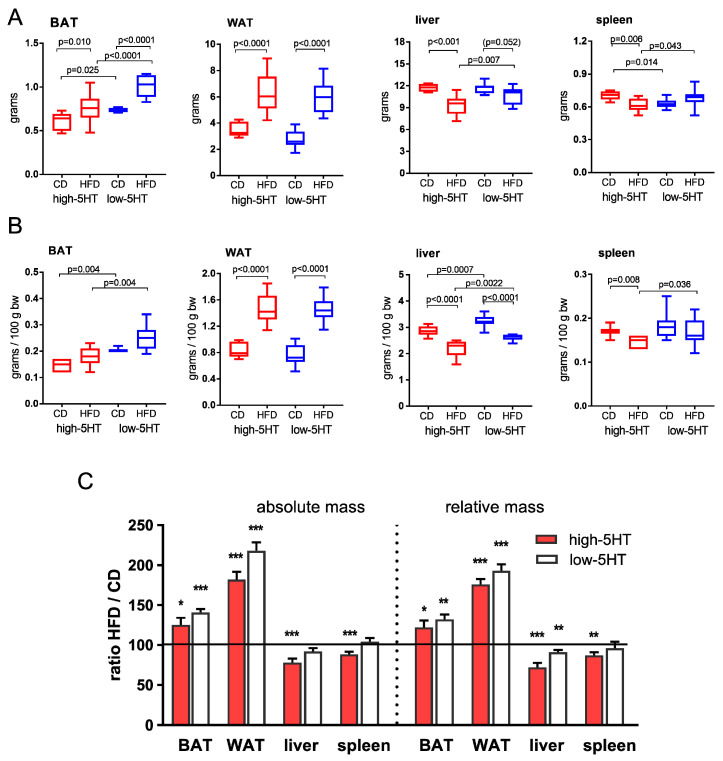
Organ mass in animals from high-5HT and low-5HT sublines at the end of 11 weeks of feeding with control diet (CD) or high–fat diet (HFD) expressed in grams per animal (**A**) or grams per body mass (**B**). Data are presented as median and min to max; N = 9 per group; *p*-values obtained by LSD or Dunn’s post-hoc test after one-way ANOVA or Kruskal-Wallis test are indicated. (**C**) Relative changes in organ mass presented as ratios between HFD-fed and CD-fed animals of each subline. * *p* < 0.05, ** *p* < 0.01, *** *p* < 0.001 (*t*-test or Mann-Whitney test, as appropriate). BAT = brown adipose tissue; WAT = gonadal white adipose tissue.

The mass of white adipose tissue (WAT) and brown adipose tissue (BAT), expressed in grams per animal (Figure 4A) or in grams per body mass (Figure 4B), was significantly higher in the HFD than CD groups of both 5HT sublines, with the increase being somewhat more pronounced in low-5HT than in high 5HT animals (HFD/CD ratio for WAT = 1.41 and 1.25, and for BAT = 2.18 and 1.82 in low-5HT and high-5HT animals, respectively) (Figure 4).

Interestingly, the mass of the other two organs examined, the liver and spleen, was significantly lower in the HFD than CD group of high-5HT subline, while in low-5HT subline only liver mass per body weight differed significantly between the HFD and CD groups. The corresponding HFD/CD ratios calculated from mass per animal were for liver = 0.92 and 0.78, and for spleen = 1.04 and 0.87 in low-5HT and high-5HT animals, respectively (Figure 4).

### 2.4. Blood Metabolic Parameters

**Figure 5:**
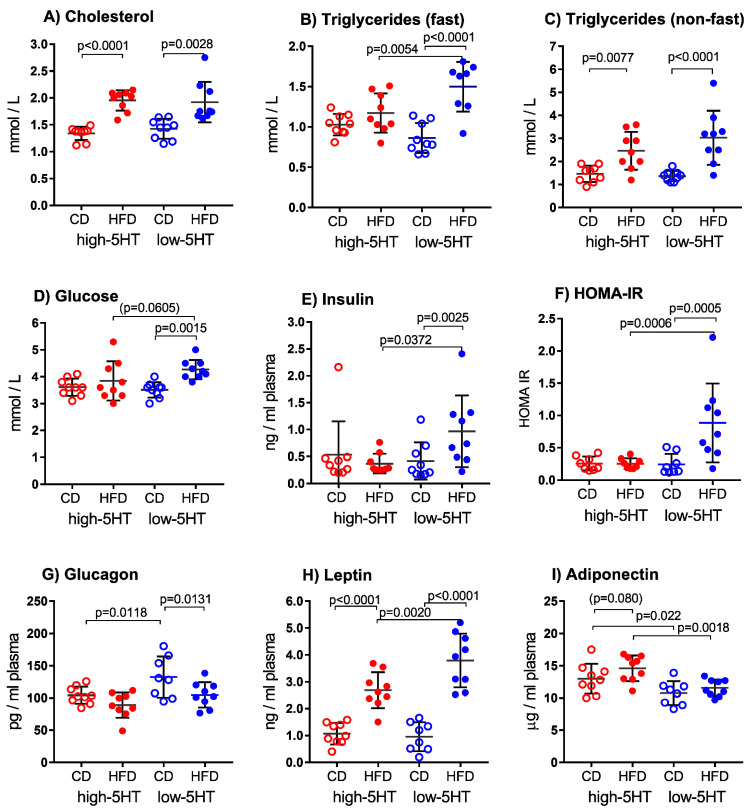
Blood biochemical parameters in animals from the high-5HT and low-5HT sublines after 4.5 (**A**,**B**) or 9–10 (**C**–**I**) weeks on control diet (CD) or high-fat diet (HFD), measured after overnight (12 h) fasting of animals (except in C). Data are presented as individual values and mean ± SD; N = 9 per group; *p*-values obtained by LSD or Dunn’s post-hoc test after one-way ANOVA or Kruskal-Wallis test, as appropriate, are indicated on figures. HOMA-IR = Homeostatic Model Assessment for Insulin Resistance, calculated from glucose and insulin fasting plasma concentrations.

As expected, feeding animals with HFD was associated with changes in blood concentrations of the blood metabolic parameters. However, the presence or magnitude of these changes was different between high-5HT and low-5HT animals (Figure 5).

Serum concentrations of lipids were determined three times, after 4.5, 9 and 10 weeks of controlled feeding. In both 5HT-sublines, total cholesterol levels were approximately 40% higher in HFD than CD groups (L-HFD/L-CD = 1.46; H-HFD/H-CD = 1.35, Figure 5A), similarly at all time points. High density lipoprotein (HDL) cholesterol levels determined after 9 weeks of controlled feeding showed similar changes as total cholesterol levels (Figure S2A). Differences in serum triglyceride (TG) concentrations between HFD- and CD-fed animals were dependent on their fasting status. Thus, when TG were measured after overnight (12 h) fasting of animals, significant increase in HFD-fed compared with CD-fed animals was observed only in low-5HT subline (L-HFD/L-CD = 1.73), but not in high-5HT subline (H-HFD/H-CD = 1.14) (Figure 5B). In non-fasting animals, a significant increase in HFD-fed animals was observed in both 5HT-sublines, although it was still higher in the low-5HT subline (L-HFD/L-CD = 2.22, H-HFD/H-CD = 1.68) (Figure 5C).

Blood glucose (Figure 5D) and insulin (Figure 5E) levels and calculated HOMA-IR (Figure 5F) were significantly higher in HFD than CD groups only in the low-5HT subline (glucose: L-HFD/L-CD = 1.21; insulin: L-HFD/L-CD = 2.31). Also, plasma glucagon (Figure 5G) levels were significantly decreased in HFD-fed animals only in the low-5HT subline (L-HFD/L-CD = 0.80).

Of the circulatory adipokines measured, leptin levels were significantly increased in HFD groups of both 5HT-sublines, but the increase was more pronounced in the low-5HT subline (L-HFD/L-CD = 3.96, H-HFD/H-CD = 2.50; Figure 5H), while adiponectin (Figure 5I) level tended to increase only in H-HFD animals (H-HFD/H-CD = 1.12, *p* = 0.08). Blood concentrations of both leptin (Figure 5H) and adiponectin (Figure 5I) were significantly different between H-HFD and L-HFD animals (leptin: H-HFD/L-HFD = 0.71; adiponectin: H-HFD/L-HFD = 1.21). Adiponectin/leptin (A/L) ratio was significantly lower in L-HFD than H-HFD animals (Figure S2B).

### 2.5. Glucose and Insulin Tolerance Test

**Figure 6:**
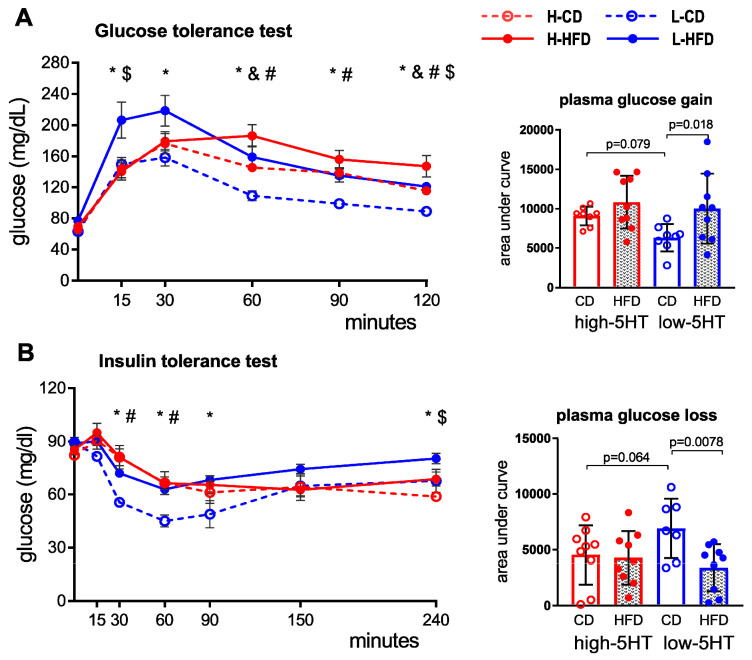
(**A**) Glucose and (**B**) insulin tolerance tests performed in animals from high-5HT and low-5HT sublines after 9 weeks on control diet (CD) or high-fat diet (HFD). The corresponding area under the curve (AUC) are given on the right. Results are presented as means ± SEM (left) or means ± SD (right, AUC) in groups of 9 animals. *p*-values obtained by LSD or Dunn’s post-hoc test after one-way ANOVA or Kruskal-Wallis test are indicated. H-CD = animals from high-5HT subline on control diet; L-CD = animals from low-5HT subline on control diet; H-HFD = animals from high-5HT subline on high-fat diet; L-HFD = animals from low-5HT subline on high-fat diet; # H-CD vs. L-CD; & H-CD vs. H-HFD; * L-CD vs. L-HFD; $ H-HFD vs. L-HFD.

To investigate the functional metabolic differences between 5HT sublines, we performed a glucose tolerance test (GTT) and an insulin tolerance test (ITT) after 4.5 and 9 weeks of controlled feeding. After 4.5 weeks, there were no significant differences between HFD-fed and CD-fed animals (not shown). After 9 weeks of controlled feeding, HFD-fed compared to CD-fed high-5HT animals showed higher blood glucose levels from 60 min after injection of glucose (Figure 6A, left), but the corresponding area under the GTT curve showed no significant differences between these groups (Figure 6A, right). Similarly, no significant differences in blood glucose levels were observed between H-HFD and H-CD groups in response to insulin loading in the ITT (Figure 6B).

On the contrary, in the low-5HT subline, the results of GTT and ITT significantly differed between HFD-fed and CD-fed animals. Specifically, L-HFD animals showed a greater increase in glucose levels in the GTT (Figure 6A, left) and a smaller decrease in glucose levels in the ITT (Figure 6B, left), compared with their L-CD counterparts. These differences persisted throughout the observation period (Figure 6, left). The corresponding area under the curves (AUC) also clearly shows that HFD affected the performance of GTT (Figure 6A, right) and ITT (Figure 6B, right) in animals of the low-5HT subline. A comparison of only HFD-fed animals from both 5HT sublines is shown in Figure S3.

### 2.6. Brown Adipose Tissue Thermogenesis

**Figure 7:**
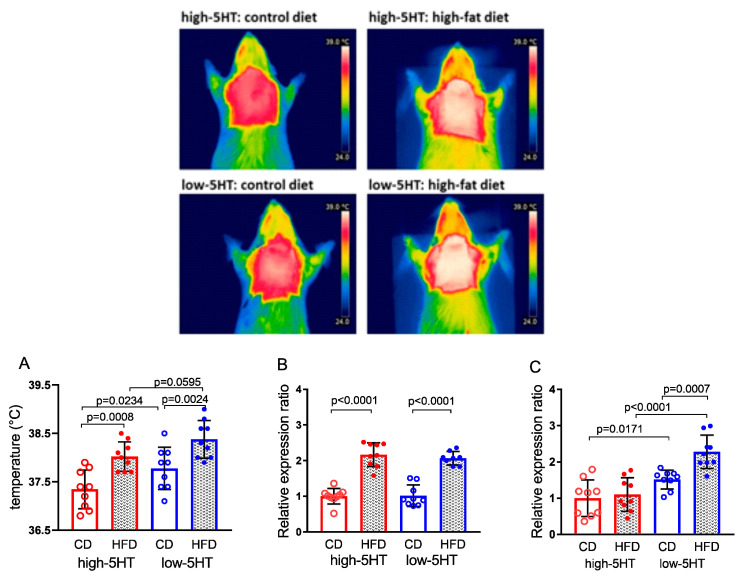
Top: Representative thermographic images of the interscapular region of animals from the 5HT sublines obtained after 8.5 weeks of control diet (CD) or high-fat diet (HFD), taken with a digital infrared camera. Bottom: (**A**) Temperature of skin over interscapular brown adipose tissue (BAT) obtained by analysis of thermographic images. (**B**,**C**) mRNA expression levels of (**B**) uncoupled protein 1 (*Ucp1*) and (**C**) fibroblast growth factor 21 (*Fgf 21*) in BAT of animals from the high-5HT and low-5HT sublines after 11 weeks of CD or HFD. Expression levels were normalized to the mean of two reference genes (*Gapdh* and *RpS29*) and are shown as relative expression ratios between groups, with high-5HT animals receiving control diet set at 1.0. Data presented as individual values and means ± SD; N = 9 rats per group; *p*-values obtained by LSD post-hoc test after one-way ANOVA.

To investigate whether there are differences in diet-induced thermogenesis between 5HT sublines, we compared the temperature of the skin over the interscapular BAT region using infrared thermography (Figure 7, top). After an 8.5-week HFD feeding, BAT thermogenesis increased similarly in both high-5HT and low-5HT sublines (by 0.68 and 0.60 °C, respectively) (Figure 7A), indicating the absence of subline-specific effect on diet-induced thermogenesis.

In both 5HT sublines, increased BAT thermogenesis was accompanied by upregulation of thermogenic uncoupling protein 1 (*Ucp1*) mRNA levels in BAT (L-HFD/L-CD = 2.03, H-HFD/H-CD = 2.16) (Figure 7B). On the other hand, mRNA levels of fibroblast growth factor 21 (*Fgf21*) in BAT were upregulated by HFD feeding only in low-5HT animals (L-HFD/L-CD = 1.51, H-HFD/H-CD = 1.10) (Figure 7C).

### 2.7. Expression Levels of Body Weight-Related Genes in Hypothalamus

To investigate which molecules/pathways underlie the observed differences between the 5HT sublines in their functional response, we compared the expression levels of various body weight- and metabolism-regulating genes in the hypothalamus and adipose tissue after 11-week controlled feeding. In hypothalamus, we focused on neuropeptides and their receptors, transcription factors and various signaling molecules.

**Figure 8:**
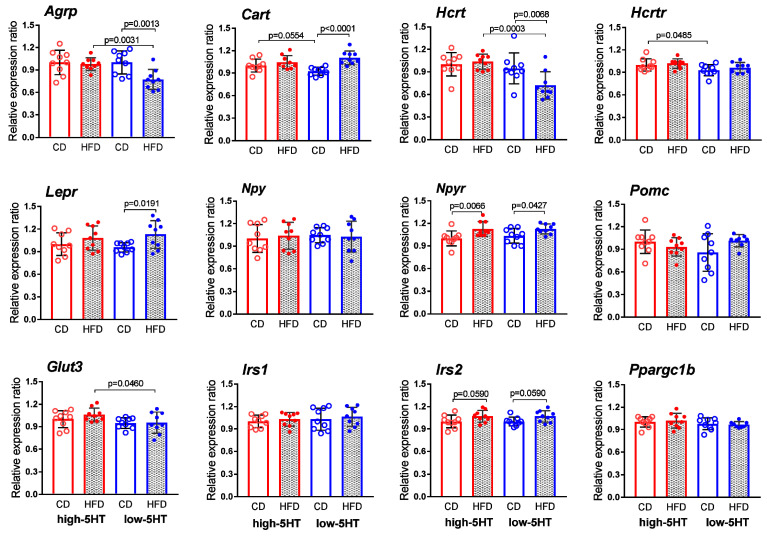
Hypothalamic mRNA expression levels of weight-related molecules, in animals from high-5HT and low-5HT sublines after 11 weeks on control diet (CD) or high-fat diet (HFD). Expression levels were normalized to the mean of two reference genes (*Actb* and *Gapdh*) and are shown as a relative expression ratio between groups, with high-5HT animals receiving the control diet set at 1.00. Data are presented as means ± SD; N = 9 per group; *p*-values obtained by LSD or Dunn’s post-hoc test after one-way ANOVA or Kruskal Wallis test, as appropriate, are indicated. Abbreviations: *Agrp* = agouti-related peptide; *Cart* = cocaine-and amphetamine-related transcript; *Glut3* = glucose transporter 3; *Hcrt* = hypocretin; *Hcrtr1*= hypocretin receptor1; *Irs1(2*) = insulin receptor substrate 1(2); *Lepr* = leptin receptor; *Npy* = neuropeptide Y; *Npyr* = neuropeptide Y receptor; *Pomc* = pro-opiomelanocortin; *Ppargc1b* = peroxisome proliferator-activated receptor gamma coactivator 1 beta.

As shown in Figure 8, among genes encoding hypothalamic neuropeptides and their receptors, significant changes in mRNA expression levels between HFD-fed and CD-fed animals were mostly present only in the low-5HT animals. In particular, L-HFD animals showed a downregulation of orexigenic peptides *Agrp* (agouti-related peptide; L-HFD/L-CD = 0.77) and *Hcrt* (hypocretin; L-HFD/L-CD = 0.76), and upregulation of anorexigenic peptide *Cart* (cocaine-and amphetamine-related transcript; L-HFD/L-CD = 1.19) as well as *Lepr* (leptin receptor; L-HFD/L-CD = 1.18), compared to L-CD group (Figure 8). The expression of *Npyr* (neuropeptide Y receptor) was significantly increased in HFD-fed animals of both 5HT sublines (L-HFD/L-CD = 1.09; H-HFD/H-CD = 1.13). There were no differences in mRNA expression of genes encoding *Hcrt1* (hypocretin receptor 1), *Npy* (neuropeptide Y) and *Pomc* (pro-opiomelanocortin) between the HFD-fed and CD-fed animals in any 5HT-subline.

Additionally, the hypothalamic mRNA expression of genes encoding receptors/transporters for peripheral signals, particularly, *Glut 3* (glucose transporter 3) and *Irs1* and *Irs2* (insulin receptor substrate 1 and 2) as well as genes encoding transcription factor *Ppargc1b* (peroxisome proliferator-activated receptor gamma coactivator 1 beta) did not differ in any of the 5HT subline between HFD and CD groups (Figure 8).

Hypothalamic mRNA levels of the 5HT transporter, an important regulator of synaptic 5HT activity, were significantly downregulated by HFD only in high-5HT animals (Figure S4).

### 2.8. Expression Levels of Body Weight-Related Genes in Adipose Tissue

**Figure 9:**
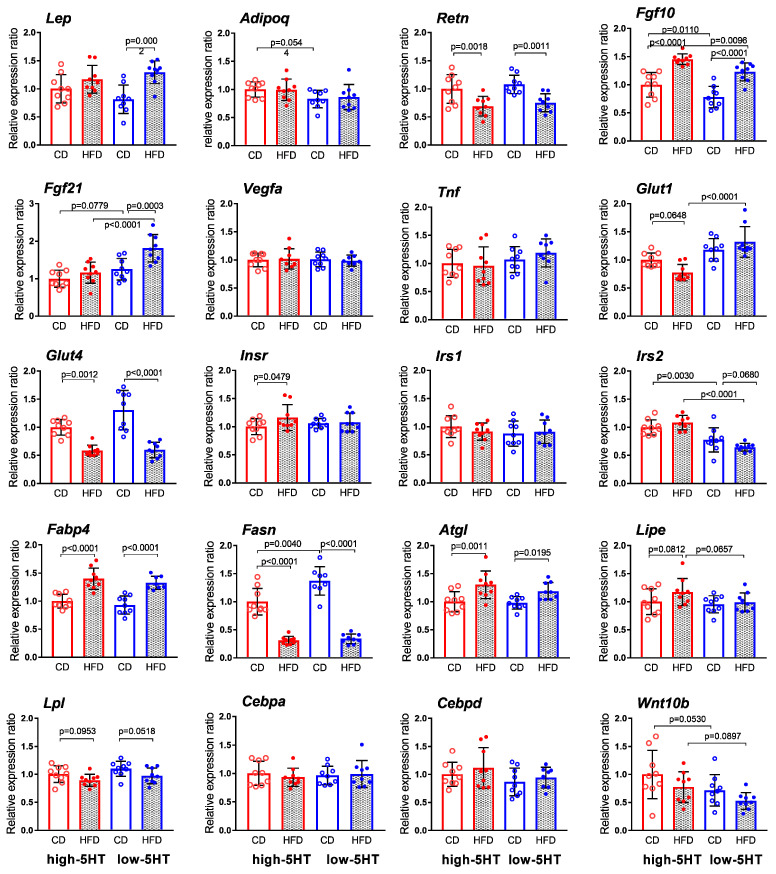
mRNA expression of selected weight-related molecules in gonadal white adipose tissue in animals from 5HT-sublines after 11 weeks on high-fat diet (HFD) or control diet (CD). Expression levels were normalized to the mean of two reference genes (Actb and Gapdh) and are shown as relative expression ratios between groups, with high-5HT animals receiving control diet set at 1.0. Data are presented as means ± SD; N = 9 rats per group; *p*-values obtained by LSD or Dunn’s post-hoc test after 1w-ANOVA or KW test, as appropriate are indicated. Abbreviations: *Adipoq* = adiponectin; *Atgl* = adipose triglyceride lipase; *Cebpa; Cebpd* = CCAAT/enhancer binding protein alfa; delta; *Fabp4* = fatty acid binding protein 4; *Fasn* = fatty acid synthase; *Fgf 10(21)* = fibroblast growth factor 10 (21); *Glut3(4)* = glucose transporter 3(4); *Insr* = insulin receptor; *Irs 1(2)* = insulin receptor substrate 1(2); *Lep* = leptin; *Lipe* = hormone sensitive lipase; Hsl; *Lpl* = lipoprotein lipase; *Retn* = resistin; *Tnf* = tumor necrosis factor; *Vegfa* = vascular endothelial growth factor A; *Wnt10b* = Wnt familiy member 10b.

The expression levels of various classes of body weight-related genes (adipokines, carbohydrate-related molecules, lipid-related molecules, growth factors and transcription factors/signaling molecules) analyzed in gonadal WAT after 11 weeks of controlled feeding are shown in Figure 9.

The expression of most of the lipid-related genes studied was equally altered in HFD-fed animals of both 5HT sublines, compared with CD-fed counterparts. In particular, mRNA levels of *Fabp4* (fatty acid binding protein 4; L-HFD/L-CD = 1.57; H-HFD/H-CD = 1.40) and *Atgl* (adipose triglyceride lipase; L-HFD/L-CD = 1.22; H-HFD/H-CD = 1.30) were upregulated, whereas mRNA levels of *Fasn* (fatty acid synthase; L-HFD/L-CD = 0.20; H-HFD/H-CD = 0.31) was downregulated. Lipoprotein lipase (*Lpl*) and hormone sensitive lipase (*Lipe, Hsl*) showed only tendency to increase and decrease, respectively, equally in both 5HT sublines.

Other genes whose expression was equally affected by HFD in both 5HT sublines were as follows: *Fgf10* (fibroblast growth factor 10) mRNA levels were significantly increased (L-HFD/L-CD = 1.57; H-HFD/H-CD = 1.45) whereas *Retn* (resistin; L-HFD/L-CD = 0.70; H-HFD/H-CD = 0.69) and *Glut4* (glucose transporter 4; L-HFD/L-CD = 0.46; H-HFD/H-CD = 0.58) mRNA levels were significantly decreased in HFD-fed animals.

No significant differences between HFD-fed and CD-fed animals of either 5HT subline were found in WAT expression levels of genes encoding transcriptional/regulatory factors involved in energy expenditure (CCAAT/enhancer binding protein a, b; Wnt family member 10b), adipocitokines (adiponectin, vascular endothelial growth factor A, tumor necrosis factor) and carbohydrate-related genes (insulin receptor substrate 1).

Probably the most important for understanding the functional metabolic differences between the 5HT sublines in response to HFD are the findings on genes that are affected by HFD in only one of the 5HT sublines. Among the carbohydrate-related genes studied, *Insr* (insulin receptor) mRNA expression was significantly increased and *Glut1* (glucose transporter 1) expression tended towards decrease only in the high-5HT animals (H-HFD/H-CD = 1.16 and 0.77 for *Insr* and *Glut1*, respectively), while *Irs2* (insulin receptor substrate 2) tended towards decreased expression only in the low-5HT animals (L-HFD/L-CD = 0.83). Of the genes encoding growth factors, *Fgf21* (fibroblast growth factor 21) expression was significantly increased only in the low-5HT subline (L-HFD/L-CD = 1.45), and of the genes encoding adipokines, *Lep* (leptin) expression was also significantly increased only in the low-5HT subline (L-HFD/L-CD =1.59).

The determination of WAT protein levels of enzymes involved in lipid synthesis and lipolysis, Fasn and Atgl, respectively, showed that HFD resulted in slight reduction of Fasn in high-5HT animals while no changes in Atgl was observed in either 5HT subline (Figure S5).

## 3. Discussion

Our previous studies on the WZ-5HT rat model have shown that constitutionally high whole-body 5HT tone is associated with increased body weight and adiposity, as well as impaired glucose and lipid metabolism [32,33,42]. In the present study, we showed that the consumption of a high-fat diet resulted in weight gain and increased insulin resistance only in the low-5HT animals, while the high-5HT animals were protected from most metabolic disturbances in the presence of obesogenic diet.

DIO rodent models, which have been repeatedly shown to have face, construct and predictive validity (reviewed in [45]), are thought to reliably reproduce the characteristics of human obesity [44,45,47]. The HFD approach is the most commonly used, showing that a diet containing more than 30% energy from fat leads to an increase in body weight and an accumulation of visceral adipose tissue [44,48]. Diets with fat proportion of 40–60% are most often used. The intervention period of 10 to 12 weeks has been recommended for the consolidation of phenotypic and metabolic features of obesity in the presence of HFD [49,50]. In our study, the increase in body weight in low-5HT animals was evident after 4 weeks of HFD feeding, while the impairment in glucose regulation, as measured by GTT and ITT, was evident after 9, but not after 4.5 weeks of HFD feeding. This is in line with reports that a longer period of dietary intervention is required for effective induction of glucose intolerance and insulin resistance in mice and rat strains [51,52].

Body weight after 11 weeks on HFD was significantly increased only in the low-5HT animals compared to the corresponding CD-fed animals. There are no standard thresholds for obesity in rodent models, but according to the commonly used criteria [44,53] only the low-5HT animals under HFD develop obesity. Given the highly overlapping values of body weight gain of individual animals from the H-HFD and L-HFD groups, our 5HT sublines cannot be strictly classified as obesity-prone or obesity-resistant. Nevertheless, it is clear that low-5HT animals are more susceptible to HFD-induced weight gain, while high-5HT animals, which have higher body weight on a standard diet, seem to be protected from further increase in body weight when consuming a high-energy diet. This is consistent with the report that peripherally injected 5HT prevents weight gain in mice fed HFD [54], but contrasts with studies showing that genetic or pharmacological inhibition of peripheral 5HT synthesis limits HFD-induced weight gain [19,25,29,55]. It should be noted that in these animal models 5HT activity was altered only in the periphery. Mice in which the function of the 5HT transporter was genetically reduced or eliminated (5HTT+/− or 5HTT−/−, respectively), did not differ from each other or from wild-type mice in the HFD-induced weight gain [56]. In this model, the differences in 5HT availability between the 5HTT genotypes are present in both the brain and the periphery, which is similar to the situation in our 5HT sublines. However, compared with our study, these authors used animals of a different species, sex, and age (female mice, 12 months old), and a different diet composition and intervention time (21.3% fat, 3 weeks), making a direct comparison with our results difficult. The reported changes in fat mass in relation to 5HT signaling in different DIO models [24,25,29,54] are also inconclusive. Our results showing a similar HFD-induced increase in fat mass in both sublines suggest that endogenous 5HT tone does not play a major role in this regard. Nevertheless, our results support the proposed role of 5HT signaling in regulating total weight gain in response to a hypercaloric diet. Recently, it was also shown that in mice fed standard chow, the upregulation of 5HT signaling promotes weight gain [57], consistent with basal phenotypic differences in our 5HT-sublines [33].

In addition to enlargement in WAT mass, HFD feeding caused an increase in interscapular BAT, as expected [58]. Consistent with our previous study [32], and in line with attenuation of BAT thermogenic activity by 5HT signaling [24,29], H-CD animals had lower BAT mass than L-CD animals. However, the HFD-induced increase in BAT mass, skin temperature above BAT, and mRNA expression of *Ucp1* (a mitochondrial protein responsible for thermogenic respiration) in BAT was similar in both 5HT sublines. This suggests that diet-induced UCP1-mediated BAT thermogenesis does not contribute to the differential body weight gain of 5HT sublines in response to a hypercaloric diet. Interestingly, HFD caused a decrease in liver weight in animals of both 5HT sublines. Due to excessive fat storage, HFD almost regularly causes an increase in liver weight, and we have no explanation for this finding. However, reduced liver size and weight were observed in HFD-fed *Htr2a* knockout (KO) mice [17] and in HFD-fed *Tph1* KO mice [29] compared with the HFD-fed wild-type mice, linking the liver growth with 5HT signaling. A direct link between physiological 5HT levels and regulation of lipid and glucose metabolism in the liver has been demonstrated [17,20,59], so it is likely that hepatic mechanisms involved in the control of metabolic homeostasis are dysregulated in our 5HT sublines.

Consistent with our previous study [32], daily food intake was higher in H-CD animals, compared with L-CD animals when measured as absolute weight and lower when normalized to body weight. However, the HFD-induced increase in total energy intake and decrease in the mass of food consumed, as well as the timing of the adjustment of food intake to the energy density of HFD, were similar in both 5HT sublines. This ruled out the possibility that food intake was responsible for the difference in weight gain between the 5HT sublines and points to their differential metabolic adaptation to excessive energy intake. It should be mentioned that CD and HFD were not adjusted for all ingredients, including sucrose content, which is the limitation of the study. A pronounced dysregulation in blood metabolic parameters and glucose homeostasis, which is expected to be triggered by excessive energy intake [60,61], was present only in the low-5HT subline. Thus, HFD consumption in low-5HT animals resulted in hyperglycemia and hyperinsulinemia, glucose intolerance and insulin resistance, as well as elevated blood leptin, cholesterol and triglycerides (TG) levels, and decreased glucagon levels. In contrast, H-HFD animals showed no changes in blood indicators of glucose metabolism and less pronounced disturbances in serum lipids, while their circulating leptin levels increased by the same extent as in low-5HT animals.

The increase in serum TG levels, which was more pronounced in the L-HFD animals may be related to their higher insulin resistance, since hypertriglyceremia, although linked to expanded adipose tissue, is also a reflection of insulin resistance [62] In the H-HFD group, serum TG levels were elevated only in fed, but not in fasting animals. These findings link constitutive 5HT activity to the acute postprandial TG (ppTG) response, which is considered a better predictor of cardiovascular disease risk and mortality than fasting TG [63]. In general, lipid metabolism regulation in WAT was similarly affected by HFD in both 5HT sublines, as shown by similar changes (increase or decrease) in the WAT expression of lipid-related genes (*Fasn*, *Atgl*, *Fabp4*, *Lipe*, *Lpl*) at both the mRNA and protein levels.

Regarding glucose homeostasis, HFD increased blood glucose and insulin levels and decreased blood glucagon levels only in the low-5HT animals, suggesting that they are unable to adapt to caloric excess, unlike the high-5HT animals. This was confirmed by significantly poorer performance of L-HFD animals in GTT and ITT compared to H-HFD animals. In addition, the temporal dynamics of changes in glucose levels over the course of GTT and ITT also differed between the 5HT sublines, with the L-HFD animals showing a faster initial response as well as faster return to baseline levels than the H-HFD animals. The dysregulation of glucose-regulating processes in various metabolic organs [64] may contribute to these differences between H-HFD and L-HFD animals in glucose tolerance.

High-5HT animals fed a standard diet exhibit glucose intolerance and insulin resistance compared to low-5HT animals [33]. Since HFD did not significantly affect the performance of high-5HT animals in GTT and ITT, they appear to have developed the coping mechanisms that prevent further deterioration of glucose homeostasis. We can hypothesize that constitutional 5HT activity contributes to the ability to withstand metabolic challenges and maintain energy homeostasis. Both insulin and glucagon are hormones produced in the pancreas and the fact that their levels were changed only in the low-5HT animals indicates a role of 5HT in the regulation of pancreatic function in response to HFD, as suggested previously [65,66]. High-5HT animals have a greater number and size of pancreatic beta-cell islets, compared to low-5HT animals [42], which may contribute to their better control of glucose homeostasis under HFD.

At the molecular level, HFD downregulated the insulin-responsive *Glut4* mRNA expression in WAT of both 5HT sublines, suggesting their similar regulation of glucose transport mediated by this transporter. On the other hand, insulin-independent *Glut1* and *Irs2* expression was significantly different between the H-HFD and L-HFD groups. These results generally suggest that both insulin-dependent and insulin-independent glucose uptake as well as signaling downstream of the insulin receptor in adipocytes may contribute to functional differences between 5HT sublines in response to HFD challenge.

While hyperinsulinemia is a common finding in HFD-fed animals [44], data on circulating glucagon concentrations are contradictory, showing increase [67], decreases [68] or no changes [69]. Several methodological issues have been proposed for these inconsistencies [67,68,69], while our results suggest that endogenous 5HT tone may influence the regulation of blood glucagon level in response to HFD. Abnormal glucagon secretion that has been shown to contribute to the development of glucose intolerance [70] may contribute to the functional differences in our 5HT-sublines.

Leptin, a hormone produced and secreted by adipose tissue, is generally increased in proportion to body fat mass [71]. However, high-5HT animals, although more obese, have similar circulating leptin levels as the low-5HT animals [33]. HFD consumption led to significant increase in circulating leptin in both 5HT sublines, but expression of *Lep* mRNA in WAT and leptin receptor (*Lepr*) mRNA in hypothalamus was upregulated only in the low-5HT animals. This suggests that HFD differentially affected leptin signaling in 5HT sublines, both centrally and peripherally. Leptin acts as an anorexigenic hormone which binds to leptin receptors on hypothalamic neurons, reducing the production of the orexigenic neuropeptides, and, consequently decreasing appetite [72]. Increased expression of hypothalamic *Lepr* in L-HFD animals may enable leptin binding and thus prevent development of central leptin resistance. These results suggest that endogenous 5HT activity may have a regulatory role in the development of leptin resistance that was shown in HFD-fed rats [73]. In addition to *Lepr*, HFD feeding increased the mRNA expression of the anorexigenic *Cart* peptide and decreased expression of the orexigenic *Agrp* and *Hcrt* peptides, again only in the low-5HT animals. The expression of Npy, the most potent natural orexigenic signal, was not altered in either HFD group, but the upregulation of *Nypr* mRNA in both HFD-fed groups suggests the overactivity of the Npy system. Taken together, the results showed that concomitant alteration of orexigenic and anorexigenic signaling pathways in HFD-fed animals were more pronounced in low-5HT subline, which potentially contributed to their susceptibility to the development of obesity under HFD.

These changes in hypothalamic gene expression could possibly be attributed to the differences in synaptic 5HT activity between the 5HT sublines. Namely, basal 5HT levels in the hypothalamus did not differ significantly between 5HT sublines [32], but high-5HT animals had higher mRNA levels of the 5HT transporter [32], which, together with other neurochemical data [32,37,38], is interpreted as enhanced synaptic 5HT activity. In this study, hypothalamic 5HT levels were not available, while expression analysis confirmed increased 5HTT mRNA levels in H-CD compared to L-CD animals and showed that HFD downregulated 5HTT expression only in the high-5HT subline. These results suggest a link between hypothalamic 5HT signaling and neuropeptide gene regulation in response to HFD, but a true understanding of this relationship requires further studies, preferably in individual hypothalamic nuclei.

Unlike leptin, blood adiponectin levels are thought to be inversely related to obesity [74] and therefore should decrease in the course of HFD consumption. This was not observed in our study, adding to the extreme complexity of adiponectin regulation in obesity and insulin resistance [75]. Furthermore, the effect of HFD feeding on fasting serum adponectin levels remains controversial in the literature, showing increases, decreases, or no changes in various DIO models [63,76,77]. Consistent with our previous study [33], H-CD animals had increased plasma adiponectin concentrations compared to L-CD animals. HFD similarly affected blood adiponectin concentration in both 5HT-sublines, resulting in significantly lower levels in L-HFD compared to H-HFD animals. As low circulating adiponectin concentrations have been associated with insulin resistance [78], this finding could potentially explain a greater metabolic deficit in low-5HT animals. A low plasma adiponectin/leptin (A/L) ratio has recently been suggested to be a marker of adiposopathy and a good predictor of insulin resistance [79]. In L-HFD animals this ratio is lower than in H-HFD animals, consistent with their higher HOMA-IR, a classic indicator of insulin resistance.

Among molecules known to be physiological regulators of body weight/energy homeostasis are several members of the Fgf family, with Fgf21 recognized as a key hormone with beneficial effects on insulin sensitivity and glucose/lipid metabolism [80,81]. While mRNA levels of *Fgf10*, which is known to be a stimulator of adipogenesis [82] were markedly up-regulated in WAT of both HFD groups, likely contributing to increased adiposity of animals in both 5HT sublines, expression of *Fgf21* was increased only in HFD-fed low-5HT-animals, in both WAT and BAT compartments. Fgf21 stimulates glucose uptake in adipocytes, improves insulin sensitivity and glycemic control, enhances BAT thermogenesis and ameliorates dyslipidemia [80,83], so its overexpression in L-HFD animals may represent an attempt to overcome the metabolic derangements. Our results support and extend our previous findings [33] that Fgf21 expression may be regulated by endogenous 5HT activity.

The susceptibility to obesity and its metabolic features in response to HFD is highly variable among strains of rats/mice [63,84], and shows great inter-individual variation [48,85]. In an attempt to better understand the genetic background for the body’s response to a high-energy diet, several underlying central and/or peripheral mechanisms have been hypothesized [44]. Studies of basal phenotypic differences in neurobiological mechanisms responsible for vulnerability to diet-induced weight gain were focused primarily on the dopaminergic and glutamatergic systems [85]. Here, we have shown that endogenous differences in the activity of the serotonergic system are an important determinant of the organism’s response to high-energy diets. The important advantage of the rat model we used is that constitutive differences in 5HT tone are present in both brain and periphery and therefore probably reflect the physiological situation in humans. In addition, just as the prevalence and metabolic consequences of human obesity differ between males and females, the rodents’ response to DIO is influenced by sex [44,86,87,88,89]. The study aimed to investigate the effects of HFD on metabolic health in females from 5HT sublines is currently underway in our laboratory. Preliminary results show that HFD-induced weight gain is more pronounced in the low-5HT subline, similar as demonstrated here for males.

In conclusion, we have shown that the body weight gain and the metabolic consequences of energy-dense diet depend, at least to some extent, on the constitutional 5HT tone. Thus, rats with constitutionally low 5HT activity gained more weight, exhibited poorer glucose control and showed more pronounced changes at the molecular level in response to HFD. On the other hand, high-5HT animals, which exhibit higher adiposity on a standard diet, appear to be “metabolically adapted” to the increased adiposity and thus develop fewer metabolic disturbances when fed a HFD. The WZ-5HT rat model, particularly its high-5HT subline, which developed less adverse metabolic outcomes on hypercaloric diets, could potentially prove useful in better understanding the conditions of metabolically healthy obesity in humans.

## 4. Materials and Methods

### 4.1. Animals

Studies were conducted on two sublines of Wistar-Zagreb 5HT (WZ-5HT) rats developed by directed breeding of animals toward extremes of platelet serotonin level (PSL) and platelet serotonin uptake (PSU) [37]. Briefly, the male and female animals with the highest and lowest platelet 5HT parameters were mated to generate high-5HT and low-5HT sublines, respectively. PSL and PSU values were determined in the offspring of each new generation and the animals with the extreme values were selected as parents for the next generation. The divergence of mean PSL values stabilized after 5–6 generations of selective breeding at approximately 70% (low-5HT subline) and 150% (high-5HT subline) of the mean value of the starting population. In this study, male animals aging 2.5 months at the initiation of the controlled feeding experiment, were used. They were housed three per cage under controlled conditions (temperature 22 ± 2 °C; humidity 55% ± 10%; 12-h light–dark cycle) and received food (standard or high-fat) and tap water ad libitum. All experiments were approved by institutional (Ruđer Bošković Institute, Zagreb, Croatia) and national (Ministry of Agriculture of the Republic of Croatia) ethical committees and were conducted in accordance with the ILAR Guide for the Care and Use of Laboratory Animals and Croatian animal protection law (NN 135/06 and 37/13).

### 4.2. Experimental Design

**Figure 10:**
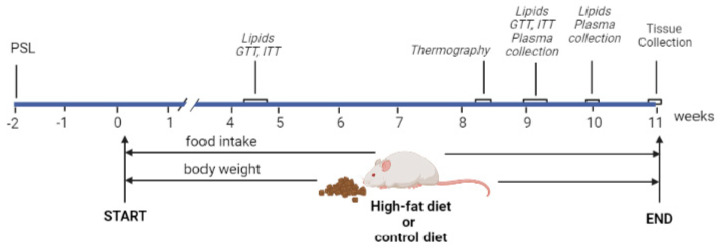
A scheme illustrating the experimental design and the timing of the analyses. Two weeks before the start of the experiment, the high-5HT and low-5HT animals were divided in two groups (N = 9 per group) and subjected to blood sampling for verification of platelet serotonin levels (PSL). Controlled feeding began when the animals were 2.5 months old and continued for the next 11 weeks with food intake and body weight monitored twice a week. All animals underwent blood glucose tolerance test (GTT), insulin tolerance test (ITT), blood sampling, and thermography at the indicated time points. At the end of the experiment, the animals were sacrificed, the organs were weighed, and tissue samples taken for further analysis. Created with Biorender.

A schematic diagram of the experimental design is shown in Figure 10. Animals from each subline were randomly divided into two experimental groups–control diet (CD) and high-fat diet (HFD) group (9 animals per group). Two weeks before the start of the controlled feeding, PSL was determined spectrophotometrically in anticoagulated blood samples taken from the jugular vein under isoflurane anesthesia (SomnoSuite anesthesia system; Kent Scientific, Torrington, CT, USA) as previously described [36]. Animals’ body weight was monitored for one week before the start of the feeding experiment. For the next 11 weeks animals from the CD group of each subline continued with a standard rodent chow diet (4RF21, 2.7 kcal/g, 6.55% kcal from fat, Mucedola, Settimo Milanese, Italy) while animals from the HFD groups were switched to a HFD (TD.06415, 4.6 kcal/g 45% kcal from fat, saturated 35% of total fat, Envigo, Indianapolis, IN, USA). Composition of CD and HFD was given in Table S1. The body weight and food intake of the animals were monitored 2 times per week during the 11-week period of controlled feeding, avoiding days when blood sampling or any other procedure was performed. At 9 am, rats were weighed and given a pre-weighed amount of food. After 24 h, the remaining food was weighed and subtracted from the amount of food given to determine the food consumed.

Blood samples for biochemical analyses were collected after 4.5, 9, and 10 weeks of controlled feeding. GTT and ITT were performed after 4.5 and 9 weeks of controlled feeding, and thermographic activity of brown adipose tissue was assessed after 8.5 weeks. After 11 weeks of controlled feeding, all animals were sacrificed by decapitation, the brain was removed and dissected, and selected organs (gonadal WAT, interscapular BAT, liver, spleen) were manually dissected and wet-weighed. A small portion of the tissues were excised and stored for further gene/protein expression analyses.

### 4.3. Biochemical Analyses of Blood Samples

Triglyceride, total cholesterol and high-density lipoprotein (HDL) cholesterol levels were measured in the serum samples using a clinical chemistry analyzer (Olympus AU480, Beckman Coulter, Brea, CA, USA). Serum samples were obtained by centrifugation (1000× *g*, 15 min) of blood samples taken from the jugular vein in plastic tubes with clot activator (BD Microtainer^®^ SST II).

The concentrations of insulin, leptin, adiponectin (Demeditec Diagnostics, Kiel, Germany) and glucagon (Elabscience, Wuhan, China) were determined in the plasma samples using a commercially available enzyme-linked immunosorbent assay (ELISA) with a sensitivity of 0.1 ng/mL (insulin), 0.081 ng/mL (adiponectin), <0.25 µg/mL (leptin) and 37.5 pg/mL (glucagon). Plasma samples were prepared by centrifugation (1500× *g*, 10 min) of blood samples taken from the jugular vein in plastic tubes containing EDTA salts (BD Vacutainer™ K2E), and were stored in aliquots at −80 °C until analyses were performed.

Glucose levels were determined in blood samples taken from an incision at the tip of the tail. Measurements were performed with a glucometer (iDIA, Hof, Germany) using the glucose oxidase method.

The Homeostatic Model Assessment for Insulin Resistance (HOMA-IR) was calculated using the following formula: fasting plasma insulin (mIU/L) * fasting blood glucose (mmol/L)/22.5.

### 4.4. GTT and ITT

For GTT, the animals were fasted for 12 h, with free access to water. Baseline blood glucose levels were measured in blood samples obtained as described in 4.3. Animals were then injected intraperitoneally with a glucose solution (20%) at a dosage of 2 g/kg body weight, and glucose levels in blood collected via the tail tip bleed were measured 15, 30, 60, 90 and 120 min after injection using a glucometer.

For ITT, which was performed two days after GTT, animals were fasted for 3 h. Baseline blood glucose was measured as described above. Animals were then injected intraperitoneally with insulin (Humulin R, Eli Lilly, Indianopolis, IN, USA) at a concentration of 0.5 units/kg body weight. Blood glucose levels were measured 15, 30, 60, 90, 150, and 240 min after injection.

### 4.5. Infrared Thermography

Infrared thermography was used to assess the thermogenic activity of interscapular BAT in animals after 8.5 weeks of controlled feeding. On the day before imaging, animals were shaved in the upper dorsal region of the scapula. On the day of thermographic imaging, they were briefly anesthetized with isofluran (SomnoSuite, Kent Scientific, Torrington, CT, USA), placed on a marble base and their paws were fixed with elastics bands to ensure the same position of all animals during imaging. Thermographic images were taken using the T335 digital infrared camera (FLIR32 Systems Inc, Wilsonville, OR, USA) with the minimum detectable temperature resolution <0.05 °C at 30 °C. For analysis of thermal images FLIR ResearchIR software and ThermoMED software, which we had previously developed [90], were used.

### 4.6. Tissue Collection for Expression Analyses

The animals were sacrificed by decapitation, and the brains were quickly removed from the skull and placed with the dorsal side on the chilled plate for dissection. The hypothalamus (cca. 20 mg/sample) was carefully removed using curved forceps and the stereotaxic atlas of the rat brain [91] as a guide, and immediately placed in RNAlater solution (Qiagen, Venlo, Netherlands) for subsequent mRNA expression analyses. Gonadal WAT (cca. 100 mg/sample) and BAT (cca 30 mg/sample) were excised and stored in RNAlater solution. In addition, WAT (cca 300 mg/sample) was excised and immediately frozen in dry ice for selected protein determination. All tissue samples were stored at −80 °C until further analysis.

### 4.7. mRNA Expression Studies

Total RNA was isolated from hypothalmus and BAT samples using the RNeasy Tissue Kit and from WAT samples using the RNeasy Lipid Tissue Kit (all from Qiagen, Germantown, MD, USA). The manufacturer’s protocol, with optional on-column DNA digestion step, was followed. Homogenisation was performed using the GentleMacS Tissue Dissociator (Miltenyi Biotec, Bergisch Gladbach, Germany). The concentration and purity of the isolated RNAs were determined spectrophotometrically (NanoDrop, Implen Gbmh, Munchen, Germany). The absorbance ratio at 260:280 nm ranged from 2.16–2.29. The integrity of the RNAs was checked using 1% agarose gel electrophoresis and Midori Green Advance staining (Nippon Genetics Europe GmbH, Dueren, Germany). All samples showed sharp 28S and 18S bands in a ratio of about 2:1.

The relative levels of specific mRNAs were determined by reverse transcription-quantitative real-time PCR (RT-qPCR) based on SYBR Green detection chemistry, as described in our previous study [33]. Sequences of primers used in qPCR are listed in Table S2.

### 4.8. Protein Expression Studies

WAT samples were homogenized in tissue protein extraction reagent (T-PER, Thermo Scientific, Waltham, MA, USA) containing protease inhibitor (Halt Protease Inhibitor Cocktail, Thermo Scientific, Waltham, MA, USA). The lysates were centrifuged (12,000× *g*, 20 min, +4 °C) and the total protein content in supernatants was determined by the Bradford method. Adipose triglyceride lipase (Atgl) and fatty acid synthase (Fasn) levels in the supernatants were determined by ELISA (Elabscience, Wuhan, China) according to the manufacturer’s protocol. The sensitivity of the assays was 0.47 ng/mL (Atgl) and 0.19 ng/mL (Fasn).

### 4.9. Statistical Analysis

Statistical analyses were performed using GraphPad Prism v8.4.3 (GraphPad Software, San Diego, CA, USA). Normality of data distribution was tested with D’Agostino–Pearson omnibus test and the presence of outliers with Grubbs test. Standard error of the ratio was calculated where appropriate. Normally distributed data were analyzed using two-tailed unpaired Student t-test for comparison of two experimental groups; for comparison of all four groups, one-way analysis of variance (1w-ANOVA) with Fisher’s least significant difference (LSD) post-hoc test was used. Non-normally distributed data were analyzed using the Mann-Whitney (MW) test for comparison of two experimental groups or Kruskal-Wallis (KW) with Dunn’s post-hoc test when compared all four groups. Total area under the curve (AUC) was calculated using the linear trapezoidal method. Results are presented as individual values and/or group means with standard deviation (SD) or standard error of the mean (SEM). Differences were considered statistically significant when *p* < 0.05. *p*-values < 0.1 were considered a trend and are shown in brackets.

## Data Availability

All data generated for this study are included in the article/Supplementary Material.

## Supplementary Materials

The following supporting information can be downloaded at: https://www.mdpi.com/article/10.3390/ijms24032169/s1.

## Abbreviations

*5HT*, serotonin; *5HTT*, membrane 5HT transporter; *Actb*, actin beta; *Adipoq*, adiponectin; *Agrp*, agouti-related peptide; *Atgl/Pnpla2*, adipose triglyceride lipase/patatin like phospholipase domain containing 2; *AUC*, area under curve; *BAT*, brown adipose tissue; *Cart*, cocaine- and amphetamine-regulated transcript; *CD*, control diet; *Cebpa*, CCAAT enhancer binding protein alpha; *Cebpd*, CCAAT/enhancer binding protein delta; *DIO*, diet-induced obesity; *ELISA*, enzyme-linked immunosorbent assay; *Fabp4*, fatty acid binding protein 4; *Fasn*, fatty acid synthase; *Fgf10(21)*, fibroblast growth factor 10(21); *Gapdh*, glyceraldehyde-3-phosphate dehydrogenase; Glut1(3,4), glucose transporter 1(3,4); *GTT*, glucose tolerance test; *Hcrt*, hypocretin neuropeptide precursor; *Hcrtr1*, hypocretin receptor 1; *HFD*, high-fat diet; *HOMA-IR*, homeostatic model assessment–insulin resistance; *Insr*, insulin receptor; *Irs1(2)*, insulin receptor substrate 1(2); *ITT*, insulin tolerance test; *Lep*, leptin; *Lepr*, leptin receptor; *Lipe/Hsl*, lipase E, hormone sensitive type; *Lpl*, lipoprotein lipase; *Npy*, neuropeptide Y; *Npyr*, neuropeptide Y receptor; *Pomc*, proopiomelanocortin; *Ppargc1b*, PPARG coactivator 1 beta; *PSL*, platelet serotonin level; *Retn*, resistin; *Rps29*, ribosomal protein S29; *RT-qPCR*, reverse transcription-quantitative polymerase chain reaction; *Sert* = serotonin transporter (5HTT); *Tnf*, tumor necrosis factor; *Ucp1*, uncoupling protein 1; *Vegfa*, ascular endothelial growth factor A; *WAT*, white adipose tissue; *Wnt10b*, wingless-type MMTV integration site family member 10B; *WZ-5HT*, Wistar-Zagreb 5HT rat.
