# Supplementary material for: Metabolic and Molecular Response to High-Fat Diet Differs between Rats with Constitutionally High and Low Serotonin Tone
Source: Int J Mol Sci. 2023 Jan 21;24(3):2169. doi: 10.3390/ijms24032169 (PMC9916796; doi:10.3390/ijms24032169)
Supplement: Supplementary file 1 [file ijms-24-02169-s001.zip › ijms-2111844-supplementary.pdf]

Supplementary material

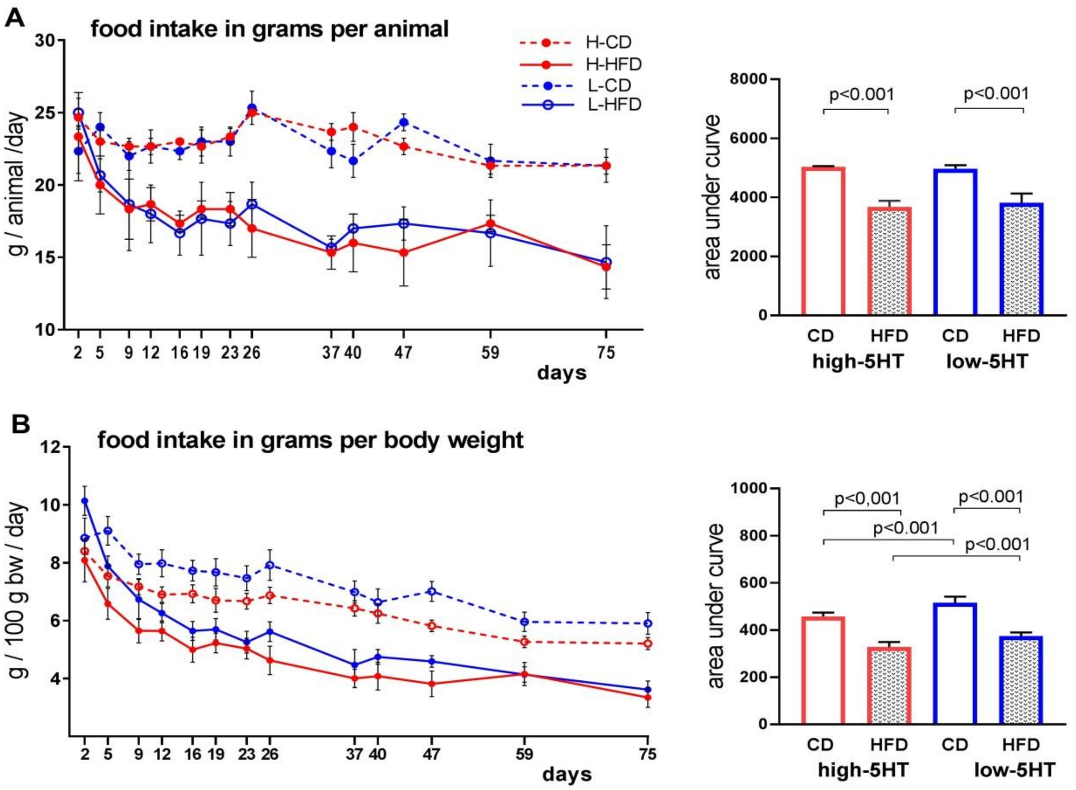

Figure S1. Daily food intake in animals from 5HT sublines over 11 weeks of feeding with control diet (CD) or high–fat diet (HFD), expressed in grams per animal (A), and grams per body weight (B). Corresponding AUC values are shown on the right. Data are presented as means ± SD; N = 9 per group; p-values obtained by LSD test after one-way ANOVA. H-CD = animals from high-5HT subline on control diet; L-CD = animals from low-5HT subline on control diet; H-HFD = animals from high-5HT subline on high-fat diet; L-HFD = animals from low-5HT subline on high-fat diet.

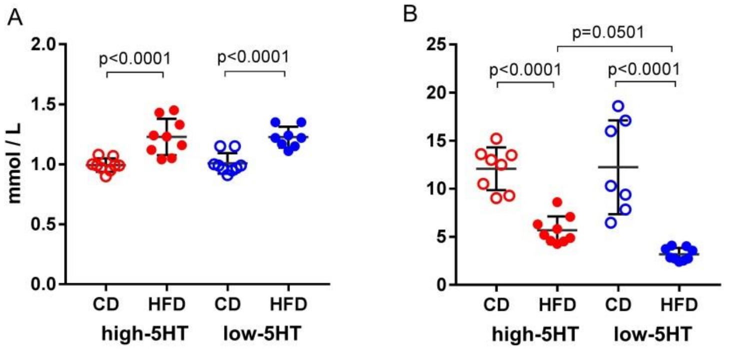

Figure S2. A) Serum concentration of high-density lipoprotein (HDL) and B) ratio between adiponectin and leptin plasma levels in high-5HT and low-5HT animals after 9 weeks of control diet (CD) or high-fat diet (HFD). Data are presented as individual values and mean ± SD; N = 9 per group; p-values obtained by LSD after one-way ANOVA are indicated on figures.

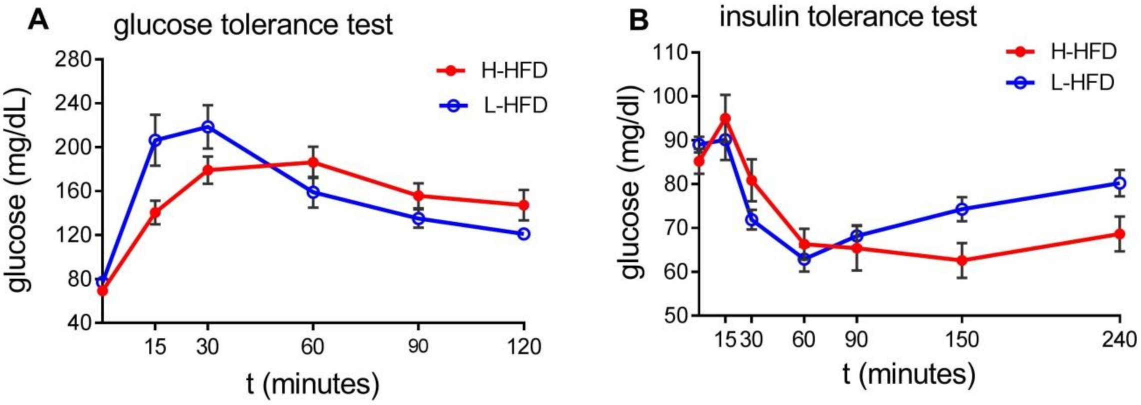

Figure S3. Comparison of glucose (A) and insulin (B) tolerance in animals of the high-5HT and low-5HT sublines after 9 weeks of feeding a high-fat diet. Results are presented as means ± SEM in groups of 9 animals. The same data are in Figure 6 in the main text.

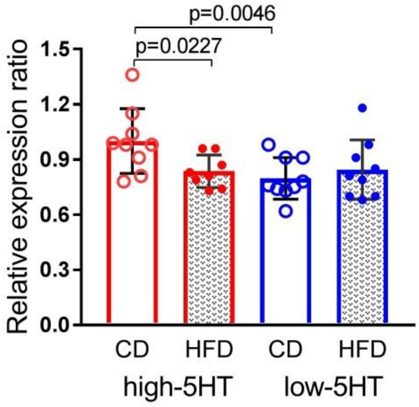

Figure S4. Hypothalamic mRNA expression levels of the serotonin transporter (*Sert*) in animals from high-5HT and low-5HT sublines after 11 weeks of control diet (CD) or high-fat diet (HFD). Expression levels were normalized to the mean of two reference genes (*Actb* and *Gapdh*) and are shown as a relative expression ratio between groups, with high-5HT animals receiving the control diet set at 1.00. Data are presented as means ± SD; N = 9 per group; p-values obtained by LSD post-hoc test after one-way ANOVA.

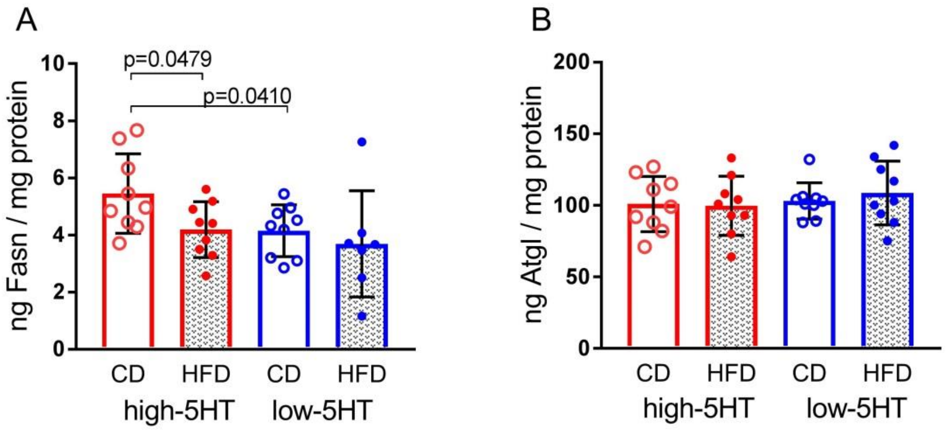

Figure S5. Concentration of A) fatty acid synthase (Fasn) and B) adipose triglyceride lipase (Atgl) in white adipose tissue of animals from the high-5HT and low-5HT sublines after 11 weeks on control diet (CD) or high-fat diet (HFD). Data are presented as individual values and mean ± SD; N = 9 per group; p-values obtained by LSD after one-way ANOVA are indicated on figures.

Table S1. Composition and energy content of control and high-fat diet

	Control diet	High-fat diet
Proteins (% by weight)	18.5	21.7
Carbohydrates (% by weight)	53.5	41.4
corn starch	42.6	8.5
sucrose	3.6	20
maltodextrin		11.5
Fats (% by weight)	3	22.7
% of total fat:		
saturated	19	35
monounsaturated	21	25
polyunsaturated	60	40
energy (Kcal/g)	2.66	4.6

Control diet: Mucedola 4RF21, Italy; High-fat diet: Envigo TD.06415 (45/Fat), USA

Table S2. Primer sequences used in RT-qPCR analysis

NCBI Symbol	Gene	Forward primer sequence	Reverse primer sequence
**Actb**	Actin beta	GCGCAAGTACTCTGTGTGGA	GGCATCATTGACGACATCGAG
**Adipoq**	Adiponectin	GAGACGCAGGTGTTCTTG	CCTACGCTGAATGCTGAG
**Agrp**	Agouti-related peptide	GCAGAGGTGCTAGATCCACAGAA	AGGACTCGTGCAGCCTTACAC
**Atgl/ Pnpla2**	Patatin like phospholipase domain containing 2	AGACTGTCTGAGCAGGTGGA	GTAGCAGAATCTCGGCATCTTCC
**Cart**	Cocaine- and amphetamine-regulated transcript	GCCAAGTCCCCATGTGTGAC	CACCCCTTCACAAGCACTTCA
**Cebpa**	CCAAT enhancer binding protein alpha	GACCATCCGCCTTGTGTGTA	CTGACATTGCACAAGGCACC
**Cebpd**	CCAAT/enhancer binding protein delta	GAATTGCTACAGTTTCTTGG	ATGCGCAGTCTCTTCCTC
**Fabp4**	Fatty acid binding protein 4	AGAAGTGGGAGTTGGCTTCG	ACTCTCTGACCGGATGACGA
**Fasn**	Fatty acid synthase	GGTAGGCTTGGTGAACTGTCTC	TCTAACTGGAAGTGACGGAAGG
**Fgf10**	Fibroblast growth factor 10	GAGATGTCCGCTGGAGAAAG	CCCCTTCTTGTTCATGGCTA
**Fgf21**	Fibroblast growth factor 21	AGGCTTTGACACCCAGGATT	ACAGATGACGACCAGGACAC
**Gapdh**	Glyceraldehyde-3-phosphate dehydrogenase	TGCCCCCATGTTTGTGATG	TGGTGGTGCAGGATGCATT
**Glut1**	Glucose transporter 1	TGGCCAAGGACACACGAATACTGA	TGGAAGAGACAGGAATGGGCGAAT
**Glut3**	Glucose transporter 3	TGGCTACAACACCGGAGTCATCAA	CTGCCAAAGCGGTTGACAAAGAGT
**Glut4**	Glucose transporter 4	ATCAACGCCCCACAGAAAGT	CCTGCCTACCCAGCCAAGT
**Hcrt**	Hypocretin neuropeptide precursor	TAGAGCCATATCCCTGCCCT	GCGAGGAGAGGGGAAAGTTAG
**Hcrtr1**	Hypocretin receptor 1	GCGCGATTATCTCTATCCGAA	AAGGCTATGAGAAACACGGCC
**Insr**	Insulin receptor	ATCTCCTGGGATTCATGCTG	TACTGGGTCCAGGGTTTGAG
**Irs1**	Insulin receptor substrate 1	GATTTAAGCACCTATGCCAG	GAATCGTGAAAGAGTTCGAG
**Irs2**	Insulin receptor substrate 2	CCACACACCTGTCCTCATTG	TAATCCGCTTTGCCAAAATC
**Lep**	Leptin	GACACCAAAACCCTCAT	CAGGGTCTGGTCCATCT
**Lepr**	Leptin receptor	CCTTGGAGGACTATGGGTGTC	GTGGCGCACAAAACAGCTTA
**Lipe/Hsl**	Lipase E, hormone sensitive type	CTCCTCATGGCTCAACTCC	ACTCCTGCGCATAGACTCC
**Lpl**	Lipoprotein lipase	TTGAGAAAGGGCTCTGCCTGAGTT	TGCTTCTCTTGGCTCTGACCTTGT
**Npy**	Neuropeptide Y	AGAGATCCAGCCCTGAGACA	TCACCACATGGAAGGGTCTT
**Npyr**	Neuropeptide Y receptor	GCTGTGGAACGTCATCAGCTA	TTGATAGATCACGAAGGGCAG
**Pomc**	Proopiomelanocortin	GAGGTTAAGGAGCAGTGACTAAGA	GTAGCAGAATCTCGGCATCTTCC
**Ppargc1b**	PPARG coactivator 1 beta	CTACCAGAGCCCACCCAGTA	CAGGATGAGGAGCCAGAACT
**Retn**	Resistin	ACTTCAGCTCCCTACTG	GTCTATGCTTCCGCACT
**Rps29**	Ribosomal protein S29	GCCAGGGTTCTCGCTCTTG	GGCACATGTTCAGCCCGTAT
**Sert/5htt**	Solute carrier family 6 member 4	TCTGAAAAGCCCCACTGGACT	TAGGACCGTGTCTTCATCAGGC
**Tnf**	Tumor necrosis factor	CATCTTCTCAAAACTCGAGTGACAA	TGGGAGTAGATAAGGTACAGCCC
**Ucp1**	Uncoupling protein 1	GGGCTGATTCCTTTTGGTCTCT	GGGTTGCACTTCGGAAGTTGT
**Vegfa**	Vascular endothelial growth factor A	CCGGTTTAAATCCTGGAGCG	TTTAACTCAAGCTGCCTCGC
**Wnt10b**	Wnt family member 10B	GTAATCACGACATGGACTTTGGAG	GCACTTCCGCTTCAGGTTTT
